# Imported aseptic meningitis due to Toscana virus infection in Austria since 2006, including a case series from 2023

**DOI:** 10.1016/j.ijid.2025.108161

**Published:** 2025-12

**Authors:** Zsòfia I. Szojka, David M. Florian, Adriana Cabal, Werner Ruppitsch, Stephan W. Aberle, Ferdinand Otto, Jeremy V. Camp

**Affiliations:** 1 Institute for Medical Microbiology & Hygiene, Division for Public Health, Austrian Agency for Health and Food Safety, Vienna, Austria; 2 ECDC fellowship Programme, Public Health Microbiology path (EUPHEM), European Centre for Disease Prevention and Control (ECDC), Stockholm, Sweden; 3 Center for Virology, National Reference Center for Arboviruses, Medical University of Vienna, Vienna, Austria; 4 Institute for Surveillance and Infectious Disease Epidemiology, Division for Public Health, Austrian Agency for Health and Food Safety, Vienna, Austria; 5 Institute of Hygiene and Medical Microbiology, Medical University Innsbruck, Innsbruck, Austria; 6 Faculty of Food Technology, Food Safety and Ecology, University of Donja Gorica, Podgorica, Montenegro; 7 Department of Neurology, Paracelsus Medical University, Christian-Doppler-Klinik, Salzburg, Austria

**Keywords:** Sandfly fever, Phlebovirus, Aseptic meningitis, Vector-borne disease, Imported cases, Toscana virus

## Abstract

**Objectives:**

Toscana virus (TOSV) is an emerging sandfly-borne virus, causing meningitis and encephalitis in Mediterranean and Middle Eastern regions. Only sporadic cases have been diagnosed in Austria over the past 20 years. We aim to identify and describe TOSV-associated aseptic meningitis (TOSV-AM) cases in Austria between 2006 and 2023.

**Methods:**

We report a series of four imported cases of TOSV-AM diagnosed in Salzburg between July and September 2023, after travel to Italy. In addition, we analyzed all TOSV cases identified by the Austrian National Reference Center (2006-2018). Diagnosis was confirmed by immunoglobulin (Ig)M/G serology. Partial N-gene Sanger sequencing was performed for three cases in total.

**Results:**

*Before* the 2023 case series, we retrospectively identified a total of six TOSV-AM cases (three male and three female; median age was 43 years [22-53]). All four 2023 patients (three male and one female; median age was 45.5 years [37-50]) presented with clinical signs of meningitis, including headache, with three reporting insect bites. Lumbar puncture confirmed the diagnosis in three cases and IgM/IgG serology confirmed infection in all cases. TOSV detection by polymerase chain reaction in serum was possible in one patient, with nucleic acid sequencing identifying a lineage B strain. Previously diagnosed case patients were identified as lineage A.

**Conclusion:**

We report a total of 10 serologically confirmed cases of TOSV-AM in Austria since 2006. Despite evidence of growing incidence of TOSV circulation in neighboring countries, this is the first well-characterized case series in Austria. Considering the recent rise in TOSV cases in endemic regions, likely driven by climate change and urbanization, imported TOSV-AM cases in Austria may increase in the future.

## Highlights

• First report of imported Toscana virus (TOSV) aseptic meningitis cases in Austria
• Ten confirmed TOSV cases since 2006, three with available sequence data
• 2023 strain from patient returning from Italy identified as lineage B TOSV
• 2023 partial sequence was identical to 2018 Swiss traveler returning from Italy
• 2015 and 2018 strains from patients returning from Italy were lineage A TOSV

## Graphical Abstract

**Figure:**
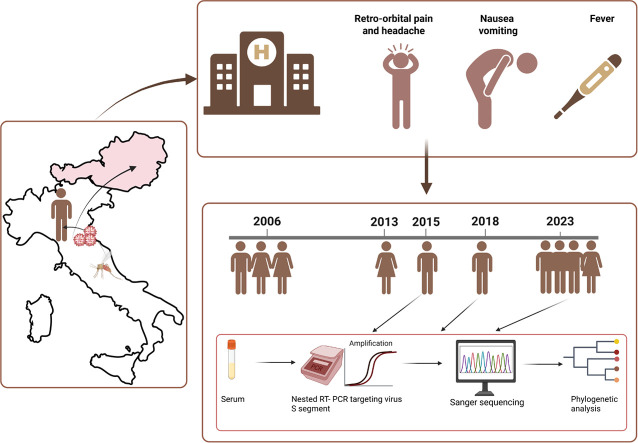

## Introduction

Toscana virus (TOSV) is a single-stranded RNA virus belonging to the sandfly-borne phleboviruses (Family *Phenuiviridae*). TOSV infections are frequently associated with neurologic disease in humans [[1], [2], [3]]. The virus was first isolated in 1971 in Italy from *Phlebotomus perniciosus* and *P. perfiliewi* [4,5]. Since then, infected vectors have been reported in several Mediterranean European countries, such as France [6], Italy [7], Malta, and Spain [8], where also more 50% of all known human cases were reported [9,10].

After an incubation period ranging from a few days to 2 weeks [11], the most common symptoms include headache, fever, nausea, vomiting, leukocytosis, Kernig’s sign, neck rigidity, and ocular manifestations including photophobia [1,9]. Atypical symptoms, such as testicular manifestations including epididymo-orchitis, testicular pain, and scrotal swelling, have been reported in five patients [12].

TOSV infection is confirmed by the detection of immunoglobulin (Ig)M and IgG antibodies [13] and by molecular detection using reverse transcription–polymerase chain reaction (RT-PCR) [14]. Due to the short period of viraemia and low viral load in the blood or cerebrospinal fluid (CSF) in the acute phase [13], direct detection of TOSV by RT-PCR is often challenging. TOSV was detected by RT-PCR approximately in 6% of patients with encephalitis and meningitis in Italy [14].

In contrast to central Italy, where the ratio of aseptic meningitis caused by TOSV is approximately 80% during warm seasons [15], aseptic meningitis in Austria is most often caused by coxsackievirus-B, echoviruses, enteroviruses (EVs) [16], tick-borne-encephalitis virus (TBEV) [17], herpes simplex virus types 1 and 2 (HSV), varicella-zoster virus (VZV) [18], West Nile virus (WNV) [19], or *Borrelia burgdorferi* [20]. In Austria, patients presenting with aseptic meningitis are not routinely tested for TOSV-AM. Elsewhere, imported cases of TOSV in travelers returning from Italy have been reported multiple times [1,[21], [22], [23]]. Two retrospective biobank studies from southern Germany reported a seroprevalence for TOSV IgM of 2% and 4%, respectively, in cases of aseptic meningitis when the causal pathogen was not identified [24,25].

Although countries such as Italy and France have reported locally acquired TOSV cases [10], and imported cases have been documented in neighboring countries [1], no cases have been reported recently in Austria. This study aimed to describe TOSV-diagnosed cases from Austria since 2006, including a recent case series in travelers returning to Austria.

## Methods

### Clinical characteristics and laboratory diagnostics of a 2023 case series

The patients were diagnosed at the Department of Neurology, Paracelsus Medical University, Christian-Doppler-Klinik in Salzburg, Austria. CSF samples were obtained from three patients at admission according to standard procedures. For these CSF samples, cell count, and red blood cells were counted; and protein, lactate, albumin, CXCL13, IgG, IgA, and IgM levels were quantified. Similar routine laboratory testing was performed from blood samples at patient admission.

CSF was tested for a range of pathogenic agents according to the clinical suspicion of the attending physician. Suspected pathogens included *Borrelia burgdorferi,* VZV, HSV, and EVs. Serum was obtained from all four patients and was screened for *Borrelia burgdorferi,* Chikungunya virus, herpesviruses (human cytomegalovirus [CMV], Epstein–Barr virus [EBV], HSV, and VZV), Dengue virus (DENV), TBEV, TOSV, and WNV. Patients with clinical suspicion were also tested for EVs, HIV*,* hepatitis C virus, influenza A and B, measles virus, mumps virus (MuV), *Mycoplasma*, and Zika virus.

For detection of TOSV, samples were analyzed at the National Reference Center for Arboviruses at the Medical University of Vienna (Vienna, Austria). Diagnosis of TOSV infection was confirmed by detection of specific IgM and IgG antibodies in serum according to the manufacturer’s instructions (Sandfly fever virus Mosaic 1 types Sicilian, Naples, Toscana, Cyprus IgG and IgM assay, EuroImmun, Luebeck, Germany). A TOSV-specific RT-qPCR (Reverse Transcription-quantitative Polymerase Chain Reaction) was performed from serum as described by Brisbarre et al*.* [26]. Virus isolation was not attempted. For sequencing and phylogenetic analysis, a nested RT-PCR was performed from serum extract using in-house developed primers targeting the virus S segment. The 713 bp amplicons were sequenced with the Sanger method and aligned to a set of reference sequences from GenBank, representing lineage A and lineage B using MAFFT (v7.515). A maximum likelihood phylogenetic tree was constructed from the nucleotide sequence alignment in IQTree2 (v2.2.0.3) using the Kimura two-parameter substitution model with four gamma categories (identified in ModelFinder) over 1000 ultrafast bootstrap iterations. The tree was rooted with the Sandfly fever Naples virus reference (NC_078079). All sequences generated in this study are available on GenBank (accession numbers PQ799302-PQ799304).

### Data sources of previously reported TOSV cases

Pre-2023 TOSV cases identified before 2023 were confirmed by the National Reference Center for Arboviruses at the Medical University of Vienna (Vienna, Austria) from June 2006 to August 2018. For inclusion in this study, cases were selected based on the presence of clinical features consistent with neuroinvasive infection and a confirmed laboratory diagnosis of TOSV. Detailed clinical laboratory analyses, including blood tests and CSF examinations, were not available.

## Results

### Case series

#### Case 1

A 43-year-old previously healthy female from Salzburg presented to the Department of Neurology in July 2023 with severe headache, mild photophobia, and myalgia for 1 week. A total of 14 days before the onset of symptoms, she returned from a 10-day trip to Tuscany, Italy. Before admission, the patient reported having fever. Upon admission, her cardiorespiratory status was stable. Physical examination revealed signs of meningeal irritation, but no other focal neurologic deficits were noted. TBEV vaccination was up to date. No skin abnormalities were observed, although the patient reported multiple insect bites during her trip (Table 1).

**Table 1:** Epidemiologic and clinical data of Toscana virus infection cases in Austria.

Case	Sex	Age	Onset	Clinical data	Travel history
**C1**	F	43	July 2023	AM, fever with severe headache, leukocytosis, photophobia, limb pain	Tuscany, Italy
**C2**	M	48	August 2023	AM, headache, epididymitis, nausea, vomiting, testicular pain and swelling	Imperia, Italy
**C3**^a^	M	50	September 2023	AM, fever with severe headache, photophobia, phonophobia, nausea, vomiting	Imperia, Italy
**C4**	M	37	September 2023	AM, severe headache, photophobia, phonophobia	Elba, Italy

AM, Aseptic meningitis.

a Partial S segment sequence available in GenBank PQ799302.

Serum laboratory test showed that serum laboratory parameters were normal, other than slightly elevated liver enzymes and mildly decreased leucocyte count. A non-contrast computed tomography scan of the brain was normal. CSF analysis showed lympho-monocytic pleocytosis with 505 cells/µl, elevated albumin quotient (24.6) indicating a disruption of the blood-brain barrier, and slightly elevated CXCL13 levels (47 pg/ml) (Supplementary Table S1 shows further serum and CSF results).

The patient was initiated on empirical intravenous treatment with acyclovir, ceftriaxone, ampicillin, and dexamethasone while awaiting serology and PCR results. Serology revealed negative IgM results for *Borrelia burgdorferi*, CMV, EBV, HSV-1/2, TBEV, and VZV; IgG antibody indexes for *Borrelia burgdorferi,* CMV, EBV, HSV, and VZV were within normal range (Supplementary Table S2); and PCR for HSV and VZV were negative.

Serum IgG for DENV was elevated (24.78 RE/ml; NR <16 RE/ml), but a test-negative IgM antibody index and absence of NS1 antigen ruled out DENV infection (Table 2). Due to negative PCR and antibody indexes, treatment with acyclovir and ceftriaxone was stopped.

**Table 2:** Results of serology test and molecular tests (PCR or RT-PCR) performed from CSF or serum samples obtained from patients with Toscana virus infections.

	Case 1		Case 2		Case 3		Case 4	
SEROLOGY TEST	IgM	IgG	IgM	IgG	IgM	IgG	IgM	IgG
**TOSV**	**Positive**	**Positive**	**Positive**	**Positive**	**Positive**	**Weakly positive**	**Positive**	**Positive**
***Borrelia burgdorferi***	Negative	Negative	Negative	Negative	Negative	Weakly positive	NT	NT
**Chikungunya virus**	Negative	Negative	Negative	Negative	Negative	Negative	Negative	Negative
**DENV**	Negative	Positive	Negative	Negative	Negative	Positive	Negative	Negative
**Dengue NS1 Antigen**	negative		NT		NT		NT	
**TBEV**	Negative	Positive	Negative	Negative	Negative	Positive	Negative	Weakly positive
**WNV**	NT	NT	Negative	Negative	Negative	Positive	Negative	Negative

MOLECULAR TESTS								

**TOSV/Serum**	Negative		Negative		**Positive**		Negative	
**TOSV/CSF**	Negative		Negative		NT		NT	
***Borrelia burgdorferi*****/CSF**	Negative		NT		NT		NT	
**Chikungunya virus/Serum**	NT		Negative		NT		Negative	
**DENV/Serum**	NT		Negative		NT		Negative	
**EVs/CSF**	Negative		NT		Negative		NT	
**HSV/CSF**	Negative		NT		Negative		NT	
**VZV/CSF**	Negative		Negative		Negative		NT	
**WNV/Serum**	Negative		Negative		Negative		Negative	

CSF, cerebrospinal fluid; DENV, dengue virus; EVs, Enteroviruses; HSV, herpes simplex virus; NS1, DENV antigen; RT-PCR, reverse transcription–polymerase chain reaction; TBEV, tick-borne encephalitis virus; TOSV, Toscana virus; WNV, West Nile virus; NT, not tested.

Because other standard viral and bacterial diagnostics remained inconclusive and considering the patient’s recent travel to an endemic area of TOSV, where she reported multiple insect bites, the presence of antibodies against TOSV were subsequently tested. The diagnosis of TOSV infection was confirmed by detection of specific IgM and IgG antibodies in serum. A TOSV-specific RT-PCR was performed with using serum and CSF, but TOSV RNA was not detected (Table 2). Cerebral magnetic resonance imaging showed unremarkable findings; in particular, there were no signs of leptomeningeal contrast enhancement and no signs of vasculitis or cerebral venous thrombosis. The patient recovered without sequelae after 20 days after symptomatic treatment and was discharged.

#### Case 2

A 48-year-old previously healthy male from Salzburg presented to the neurology department in August 2023 with nausea, vomiting, headache lasting 1 day, and testicular pain persisting for 5 days after returning from a trip to Imperia, Italy. Upon admission, the patient had stable cardiorespiratory functions and no fever. Neurologic examination revealed signs of meningeal irritation, with absence of other focal neurologic deficits. No skin abnormalities were observed. A tender and swollen right testicle was noted (Table 1).

The serum laboratory tests revealed slightly elevated liver enzymes, elevated leucocyte count, but other parameters were normal (see Supplementary Table S1). Contrast computer tomography (CCT) before lumbar puncture was normal. CSF parameters indicated an impaired blood-brain barrier function (albumin quotient of 20.8), with lympho-monocytic pleocytosis of 925 cells/µL and slightly elevated CXCL13 level (163 pg/mL) (Supplementary Table S1 shows further serum and CSF results).

The patient was started on empirical treatment with acyclovir and ceftriaxone while awaiting serology and PCR results.

Antibody testing showed serum was negative for IgM against *Borrelia burgdorferi*, Chikungunya virus, DENV, TBEV, and WNV. HIV and hepatitis C virus testing were negative. Confirmatory PCR tests excluded infection of Chikungunya virus, DENV, WNV, and Zika virus. The IgG antibody indexes for *Borrelia burgdorferi*, CMV, EBV, TBEV, and VZV were all within normal range (Supplementary Table S2). Treatment with acyclovir and ceftriaxone was stopped after negative PCR and serology.

Due to the reported travel history of the patient, a serologic test for TOSV was performed. Serologic tests for TOSV-specific IgM and IgG were positive (Table 2). An RT-PCR specific to TOSV was negative (Table 2). After symptomatic treatment, the patient recovered and was discharged 7 days after admission without sequelae.

#### Case 3

A previously healthy 50-year-old man presented to the neurology department in September 2023 with a 24-hour history of holocephalic headache, nausea, vomiting, photophobia, and phonophobia. The patient had returned from Imperia, Italy to Austria from a vacation before admission. He reported multiple insect bites during his stay in Italy. On physical examination, the patient showed signs of meningeal irritation (positive Brudzinski sign); no rash was observed.

The serum laboratory tests showed normal liver enzyme levels, alongside elevated leukocyte, neutrophilic granulocyte, and monocyte counts. CCT before lumbar puncture was normal. CSF analysis showed impaired blood-brain barrier function (albumin quotient 10.3) and mild lympho-monocytic pleocytosis with 10 cells/µL, along with elevated CXCL13 level (26 pg/mL). IgG antibody indexes for *Borrelia burgdorferi*, CMV, EBV, HSV-1/2, TBE, and VZV were all within the normal range (Supplementary Table S1 shows further serum and CSF results).

The patient was started on an empirical treatment with acyclovir and ceftriaxone.

Serum was negative for IgM against *Borrelia burgdorferi*, Chikungunya virus, CMV, Coxsackievirus, DENV, EBV, EVs, HSV, Influenza A and B, Measles, MuV, Mycoplasma, TBEV, VZV, and WNV. IgG antibody indexes against the selected pathogens were all within the normal range (Supplementary Table S2). After negative PCR for HSV and VZV empirical treatment was stopped.

**Figure 1:**
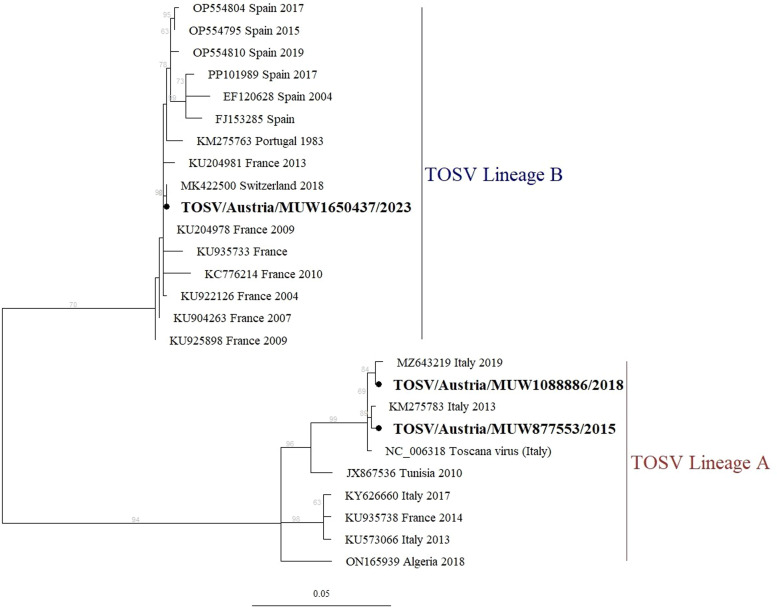
Maximum likelihood tree of the partial S segment of TOSV identified from three patients returning to Austria after travel.

Serologic testing for TOSV revealed positive IgM and weakly positive IgG reactions (Table 2). TOSV infection was confirmed by positive RT-PCR (Table 2). Sequence analysis of the partial N-gene revealed a TOSV type B strain identical to a strain found in 2018 in a Swiss traveler who returned from Imperia, Italy [27] (Figure 1). After symptomatic treatment, the patient recovered without sequelae and was discharged 2 days after admission.

TOSV, Toscana phlebovirus.

The 713 bp sequences from Austria (indicated by dots) were aligned to a set of reference sequences from GenBank representing lineage A and lineage B (clades indicated) using MAFFT. Reference sequences are labeled by GenBank accession number, country, and year of description. The tree was constructed in IQTree2 (v2.2.0.3) using the Kimura two-parameter substitution model with four gamma categories (identified in ModelFinder) over 1000 ultrafast bootstrap iterations (percent bootstrap support shown in gray text beside branches). The tree was rooted with the Sandfly fever Naples virus reference (NC_078079; not shown). All sequences generated in this study are available on GenBank (accession numbers PQ799302-PQ799304).

#### Case 4

A 37-year-old man with no past medical history presented to the outpatient clinic of the neurology department in September 2023 with a 24-hour history of frontal headache with fever, photophobia, and phonophobia, without nausea and vomiting. The patient had returned from a vacation in Elba Island, Italy 1 week before admission and reported multiple insect bites during his stay.

Leukocyte levels were normal at 9.43 G/l, with a slightly elevated level of neutrophil granulocytes (78.1%) and a decreased level of lymphocytes (14.1%). CCT scan was normal, but the patient refused the recommended lumbar puncture, although the clinical presentation indicated meningitis.

Serum was negative for IgM against Chikungunya virus, CMV, DENV, EBV, EVs, HSV, measles virus, MuV, TBEV, WNV, and VZV (Table 2).

Serology for TOSV revealed positive reactions for IgM and IgG and negative TOSV RT-PCR (Table 2). Because the patient declined inpatient admission, he was discharged home with symptomatic treatment.

### Previously recorded TOSV cases in Austria

Before the summer of 2023, a limited number of TOSV infections had been documented in Austria. Between 2006 and 2023, six imported TOSV cases were identified, consisting of three male and three female patients, with an age range of 32-53 years (median age 43 years) (Table 3).

**Table 3:** Epidemiologic and clinical data of TOSV infection cases in Austria before 2023.

Case	Sex	Age	Onset	Clinical data	Travel history
**SC1**	F	32	June 2006	Severe headache, photophobia, nausea, vomiting	Naples, Italy
**SC2**	F	22	July 2006	AM, fever with severe retrobulbar headache, photophobia, nausea,	Tuscany, Italy
**SC3**	M	46	August 2006	AM, fever with severe headache, backpain	Tuscany, Italy
**SC4**	F	40	September 2013	Fever and severe headache	Elba Island, Italy
**SC5**^a^	M	53	August 2015	AM, fever with severe headache, nausea	Elba Island, Italy
**SC6**^a^	M	49	August 2018	Fever and severe headache	Elba Island, Italy

AM, Aseptic meningitis; SC, Sporadic Case.

a Partial S segment sequence available in GenBank PQ799303 and PQ799304.

Three of the cases were reported in 2006 and one each in 2013, 2015, and 2018. All infections were reported after travels to Italy: three occurred after visits to Elba, two after returning from Tuscany, and one case after a visit near Naples.

All patients presented with headache (n = 6). Five patients also experienced fever and two experienced photophobia. Three patients were diagnosed with aseptic meningitis based on clinical symptoms or by lumbar puncture. TOSV was detected by PCR in the cases from years 2015 and 2018, both with travel history to Elba Island. The sequence analysis of the partial N-gene (PQ799303 and PQ799304, respectively) revealed that the patients were infected with a TOSV lineage A that was similar to strains identified in cases linked to Elba Island [28,29] (KM275783 and MZ643219, respectively) (Figure 1).

## Discussion

Aseptic meningitis due to imported TOSV infection in Austria is rare. Retrospective data from biobank studies from southern Germany, geographically close to Salzburg, also support a low prevalence of TOSV infection in patients with aseptic meningitis without an identified causal pathogen [24,25]. In contrast, seroprevalence of TOSV in central Italy is approximately 25% [30], and approximately 80% of cases of meningoencephalitis in central Italy were reported as TOSV infection [15], indicating a higher risk of exposure to TOSV for visitors to this region of Italy during warm months. Moreover, a recent literature review found that 77% of the TOSV cases reported between 1985 and 2023 occurred in Italy [12]. Nevertheless, if not specifically tested for, TOSV infection can easily be missed because it usually has a self-limiting, benign course.

During a relatively short period of 3 summer months (July-September 2023), four patients were serologically diagnosed with aseptic meningitis due to TOSV infection after a visit to highly endemic regions. The reported median incubation time of TOSV infection is 12 days [31]. Three of the four cases had an incubation period ranging from a few days to 10 days. In contrast, the incubation period for case 3 was reported as 3 weeks before admission. However, given the patient’s account of multiple insect bites, it is more plausible that the reported duration reflects an anamnestic inaccuracy rather than a genuinely extended incubation period.

Three of the four patients recalled insect bites during their stay in Italy. All patients were tested for TOSV virus in serum (n = 4) and two in CSF, but TOSV RNA was only detected in serum of one patient (case 3), which is consistent with the literature [32,33]. Sequencing of TOSV from this positive sample case clearly placed it within lineage B, which is composed of viral strains previously reported from France, Portugal, and Spain. In fact, this sequence showed 100% nucleotide identity to a strain characterized from a traveler living in Switzerland returning from vacation to the same region of Italy (Imperia) [27], which borders France, where TOSV lineage B is known to circulate (compared with reference strains from France in Figure 1). The expansion of lineage B TOSV may explain the close phylogenetic relationship observed between the strain from case 3 and the French isolates collected between 2004 and 2010. In contrast, sequencing of the previously reported TOSV infections in Austria from 2015 and 2018 were found to belong to lineage A. Historically, TOSV lineage A is associated with Italy and surrounding Mediterranean countries [1,34]. The phylogenetic analysis and sequence similarity of our strains provided excellent geographic correlation to previously reported strains (on the island of Elba) [28,29]. This suggests a co-circulation of two lineages of TOSV in northwestern Italy.

All four patients from the 2023 case series presented with clinical signs of meningitis, and, in three of the cases, the diagnosis was confirmed by lumbar puncture. The patients experienced the commonly observed symptoms of TOSV infections that include headache and fever (over 90%), meningitis (84%), nausea (74-81%), and ocular manifestations such as photophobia (34-54%) [1,35].

Interestingly, one of our patients (case 2) presented with testicular pain and a swollen scrotum that manifested before meningitis, which is rarely found in TOSV infection. This manifestation has previously been reported in four cases [27,28,[36], [37], [38], [39]]. In contrast to the reported cases of TOSV with testicular manifestation, which occurred in younger patients aged 16-29 years, [12], we report a patient who was nearly 50 years old at the time of infection. One previous study has shown that testicular manifestations of TOSV can lead to coagulative necrosis of the seminiferous tubules, affecting Sertoli and germ cells, as well as mild to intense tubular fibrosis and blockage of spermatogenesis [36]. All other reported cases of TOSV with testicular manifestation showed favorable outcomes, with physical improvements, including relief from testicular pain, occurring within a few days of diagnosis [27,37,38]. Of note, even if the outcome of this rare manifestation is favorable, viral RNA can be detected in spermatozoa and round seminal cells 17 days after the onset of symptoms. In addition, viral RNA has been found in seminal fluid up to 2 months after possible exposure. This suggests that spermatozoa and round seminal cells could serve as potential targets for TOSV infection, potentially contributing to observed sperm abnormalities [28]. Although it is a rare clinical manifestation, clinicians should specifically ask patients for testicular symptoms in case of TOSV infection and initiate urological follow-up.

Arboviruses such as WNV, Crimean–Congo hemorrhagic fever virus, TBEV, and TOSV, along with their vectors, have expanded their geographical range in Europe [40]. In 2022-2023, the incidence of neuroinvasive TOSV cases in Italy was increased by 2.6-fold compared with the incidence detected in the previous 5 years, which might be related to extreme climate anomalies in the Mediterranean region [31]. It was previously shown that, in addition to the increase in population density, climate change has also been a critical driver behind the heightened risk of emerging mosquito-borne pathogens in Europe, such as WNV [41].

Although millions of tourists visit the Mediterranean Basin annually and TOSV infections are on the rise in the region, TOSV is still rarely identified as a cause of aseptic meningitis in travelers returning to Austria and other countries: from January 1, 1971 to June 30, 2023, a total of 33 imported cases of TOSV infection were documented across eight countries [1,35]. Italy has been identified as the primary source of these cases, with the majority of imported infections identified in Germany (10 cases), followed by Switzerland (four cases), the United States (four cases), the United Kingdom (two cases), and the Netherlands (two cases). Isolated cases have also been reported in France (one case), Denmark (one case), and Australia (one case) [35].

Our findings provide evidence for the concurrent circulation of TOSV lineages A and B in Italy. This expands upon previous reports indicating the predominance of lineage B in Croatia, France, Morocco, Portugal, Spain, and Turkey and lineage A in Algeria, France, Italy, Tunisia, and Turkey [1]. Thus, the detection of both lineages within the same region—north Italy—suggest potential co-circulation dynamics and highlight the intricate epidemiologic dynamics of TOSV in Italy.

## Conclusion

The described case series of TOSV-associated meningitis highlights the importance of considering Toscana virus in the differential diagnosis of patients with aseptic meningitis, especially when patients report previous visits to endemic areas.

## Declaration of competing interest

The authors have no competing interests to declare.

## Appendix Supplementary materials

Image, application 1
