# Supplementary material for: Imported aseptic meningitis due to Toscana virus infection in Austria since 2006, including a case series from 2023
Source: Int J Infect Dis. 2025 Dec;161:None. doi: 10.1016/j.ijid.2025.108161 (PMC12657270; doi:10.1016/j.ijid.2025.108161)
Supplement: Supplementary file 1 [file mmc1.docx]

**Supplementary material**

This supplementary material is hosted by International Journal of Infectious Diseases as supporting information alongside the article “**Imported aseptic meningitis due to Toscana virus infection in Austria since 2006, including a case series from 2023**“ on behalf of the authors, who remain responsible for the accuracy and appropriateness of the content. The same standards for ethics, copyright, attributions and permissions as for the article apply.

***Laboratory Diagnosis of 2023 case series (Case 1, Case 2 and Case 3)***

***Case 1***

Serum laboratory tests of case 1 revealed mildly elevated levels of glutamate oxaloacetate transaminase (GOT) at 36 U/L and glutamate pyruvate transaminase (GPT) at 45 U/L, along with an increased lactate dehydrogenase (LDH) (>250 U/L), Gamma-glutamyl Transferase (GGT) at 71 U/L, pancreas-specific amylase (P-AMY) at 56 U/L, and normal C-reactive protein (CRP) levels (0.4 mg/dL) levels. Leukocyte count was normal at 9.11 G/L, but there was a mild elevation in neutrophil granulocytes (78.1%) and a decrease in lymphocytes (16.5%).

Cerebrospinal fluid (CSF) analysis revealed normal glucose levels, elevated lactate (3 mmol/L), and increased levels of total protein (144 mg/dL), albumin (113 mg/dL), IgG (13.20 mg/dL), IgA (2.31 mg/dL), and IgM (1.26 mg/dL) (see Supplementary Table S1).

***Case 2***

The serum laboratory tests revealed normal levels of GOT (14 U/L), GPT (26 U/L), along with an increased LDH level (>250 U/L), slightly elevated CRP (0.7 mg/dL), GGT (71 U/L), and Ferritin (241 μg/L) levels. Leukocyte levels were elevated at 15.05 G/L, with a slightly elevated levels of neutrophil granulocytes (81.2%) and monocytes (0.82 G/L) and a decrease in levels of lymphocytes (13%).

CSF analysis showed elevated lactate (4 mmol/L), increased total protein (101 mg/dL) and albumin (82 mg/dL) levels, with elevated levels of IgG (9.46 mg/dL) and IgA (1.27 mg/dL), and normal IgM (0.28 mg/dl) level (see Supplementary Table S1).

Additionally, paraneoplastic antibodies (anti-Hu, anti-Yo, anti-Ri and anti-Ma, Kelch-like Protein 11(KLH11)) were negative.

***Case 3***

Blood test showed elevated leukocyte count at 11.1 G/L, with a slightly elevated neutrophilic granulocytes (86.2%) and monocytes (2.7 G/L) and a decrease in levels of lymphocytes (11.1%) (see Supplementary Table S1).

CSF analysis revealed normal levels of glucose (62 mg/dL), lactate (2.5 mmol/L), and albumin (41.6 mg/dL); normal levels of IgG (5.06 mg/dL), and IgM (0.207 mg/dL), with increased levels of total protein (60 mg/dL) and IgA (0.94 mg/dL).

**Table S1.** Laboratory findings of TOSV cases, 2023, Austria.

|  | | **Normal ranges** | **Case 1** | **Case 2** | **Case 3** | **Case 4** |
| --- | --- | --- | --- | --- | --- | --- |
| **CSF analysis** | |  |  |  |  |  |
| Cells /µl | | 0-4 | 505 | 925 | 10 | NT |
| RBC count /µl | | 0 | 1 | 0 | 0 | NT |
| Proteins (mg/dl) | | 3-50 | 144 | 101 | 60 | NT |
| Lac (mmol/l) | | < 2.2 | 3.0 | 4.0 | 2.5 | NT |
| Alb (mg/dl) | | 13.9-50.0 | 113.0 | 82.0 | 41.6 | NT |
| Alb quotient | | < 8.0 | 24.6 | 20.8 | 10.3 | NT |
| CXCL13 (pg/ml) | | 0-250 | 47.0 | 163.0 | 26.0 | NT |
| IgG (mg/dl) | | 0.48-5.86 | 13.2 | 9.46 | 5.06 | NT |
| IgA (mg/dl) | | 0-0.30 | 2.31 | 1.27 | 0.94 | NT |
| IgM (mg/dl) | | 0-0.50 | 1.26 | 0.283 | 0.207 | NT |
| **Blood tests (first evaluation)** | | | | | | |
| WBCs (G/L) | | 3.5-9.8 | 9.11 | 15.05 | 3.77 | 9.43 |
| RBCs (T/L) | | 4.-5.1 | 4.9 | 5.5 | 4.7 | 5.4 |
| Hb (g/dL) | | 12-16 | 15.0 | 18.0 | 14.4 | 16 |
| PLTs abs. (G/L) | 140-400 | | 283 | 309 | 159 | 233 |
| LDH (U/L) | | 135-225 | >250 | >250 | 150 | 130 |
| Cr (mg/dL) | | 0.7-1.3 | 0.65 | 0.91 | 0.90 | 0.93 |
| CRP (mg/dL) | | <0.6 | 0.4 | 0.7 | 0.2 | 3.5 |
| GOT/GPT (U/L) | | 10-35/10-35 | 36/45 | 14/26 | 17/13 | 11.0/20.0 |
| GGT (U/L) | | 5-39 | 71.0 | 71.0 | 22.0 | 49.0 |
| P-AMY (U/L) | | 13-53 | 56.0 | 15.0 | 25.0 | 15.0 |
| Neutrophil % | | 40-75 | 78.1 | 81.2 | 86.2 | 78.1 |
| Lymphocyte % | | 18-48 | 16.5 | 13.0 | 11.1 | 14.1 |
| Monocyte % | | 4-11 | 4.7 | 5.4 | 2.7 | 6.3 |
| Monocyte abs. (G/L) | 0.2-0.6 | | 0.43 | 0.82 | 0.1 | 0.59 |
| Lymphocyte abs. (10^9^/L) | | 1.0-2.9 | 1.50 | 1.95 | 0.42 | 1.33 |
| Neutrophil abs. (G/L) | 1.6-7.10 | | 7.11 | 12.23 | 3.25 | 7.37 |

Alb: Albumin; CRP: C-reactive protein; CSF: cerebrospinal fluid; Cr: Creatinine; CXCL13: Chemokine (C-X-C motif) ligand 13; IgA, IgG, IgM: Immunoglobulin A, G, M; GGT: Gamma-glutamyl Transferase; GOT: Glutamate oxaloacetate transaminase; GPT: glutamate pyruvate transaminase; Hb: Haemoglobin; Lac: Lactate; LDH: lactate dehydrogenase; P-AMY: pancreas-specific amylase; PLTs: Platelets; RBCs: Red blood cells; WBCs: White blood cell; abs. = absolute counts; *NT: not tested*

***Table S2.*** IgG antibody indices tested at admission of patients in the case series, 2023.

| Pathogen | Normal ranges | Case 1 | Case 2 | Case 3 | Case 4 |
| --- | --- | --- | --- | --- | --- |
| *Borrelia burgdorferi* | 0.1–0.5 | UD | UD | 0.7 | NT |
| CMV | 0–1.5 | 1 | 1 | UD | NT |
| EBV | 0–1.5 | 1.2 | 0.9 | 0.8 | NT |
| HSV | 0–1.5 | 1.3 | NT | 0.8 | NT |
| TBEV | 0–1.5 | 1.2 | UD | 0.9 | NT |
| VZV | 0–1.5 | 1.1 | 0.8 | 0.9 | NT |

Cytomegalovirus: CMV; Epstein–Barr virus: EBV; HSV: Herpes simplex virus: HSV; TBEV: Tick-borne encephalitis virus; varicella-zoster virus: VZV; *NT: not tested; UD: undetectable*
